# Systemic Antibiotic Prophylaxis Adjunctive to Surgical Reconstructive Peri‐Implantitis Treatment: A Retrospective Study

**DOI:** 10.1111/cid.13429

**Published:** 2024-12-15

**Authors:** Ausra Ramanauskaite, Ioanna Saltzer, Ninad Padhye, Amira Begic, Karina Obreja, Iulia Dahmer, Frank Schwarz

**Affiliations:** ^1^ Department of Oral Surgery and Implantology Goethe‐University Frankfurt, Carolinum Frankfurt Germany; ^2^ Faculty of Medicine, Institute of Biostatistics and Mathematical Modelling Goethe University Frankfurt am Main Germany

**Keywords:** dental implants, surgical reconstructive peri‐implantitis treatment, systemic antibiotics

## Abstract

**Aims:**

To evaluate the clinical efficacy of oral systemic antibiotic prophylaxis administered along with the surgical reconstructive peri‐implantitis treatment.

**Methods:**

A total of 49 patients exhibiting 70 implants diagnosed with peri‐implantitis underwent a surgical reconstructive peri‐implantitis treatment. Of them, 27 patients (38 implants) received a single preoperative shot of antibiotics (2 g amoxicillin; Pre‐op), 12 patients (19 implants) were prescribed with postoperative antibiotics for 3 days (500 mg amoxicillin, 3 x day, Post‐op), and the remaining 10 patients (13 implants) did not receive any systemic antibiotics (No‐Ab). Mean probing depth values (mean PDs; primary outcome), bleeding on probing (BOP), plaque (PI), suppuration (Sup), and deepest PDs values (max PD) were assessed prior to surgery (baseline), after 6 and 12 months. To assess the differences in changes in the clinical parameters, and disease resolution (PD ≤ 5 mm, ≤ 1 BOP site and no Sup) among the groups, logistic regression analyses were performed.

**Results:**

After 12 months, the mean PD reduction amounted to −1.74 ± 1.56 mm, −1.91 ± 1.88 mm, and −1.13 ± 1.05 mm in the No‐Ab, Pre‐op, and Post‐op groups, respectively, with no significant difference detected among the groups. The BOP was reduced in 60%, 59.3%, and 83.3% of the patients after 12 months in the No‐Ab, Pre‐op, and Post‐op groups, respectively, with no significant differences among them. The PI, Sup and max PD reductions were comparable among the groups. Disease resolution after 12 months was established in 61.5%, 73.7%, and 89.5% of patients in the No‐Ab, Pre‐op, and Post‐op groups (No‐Ab vs. Pre‐op: *p* = 0.10, No‐Ab vs. Post‐op: *p* = 0.40, Pre‐op vs. Post‐op: *p* = 0.84).

**Conclusion:**

Systemic antibiotic prophylaxis did not improve the clinical outcomes of surgical reconstructive peri‐implantitis treatment.

## 1 Introduction

Peri‐implantitis defines an inflammatory disease affecting soft‐ and hard‐tissue surrounding a dental implant [1]. Although bacterial‐plaque plays a major role in the etiology of the pathology, there is strong evidence that there is an increased risk of developing peri‐implantitis in patients who have a history of chronic periodontitis, poor plaque control skills, and no regular maintenance care after implant therapy [2]. Peri‐implantitis‐affected sites feature bleeding on probing (BOP), increased probing depth values (PDs), with or without subsequent suppuration (Sup)/soft‐tissue recession, and progressive marginal bone loss [1]. Based on meta‐analyses, the mean estimated values for peri‐implantitis prevalence ranges between 20% and 22% at patient‐level [3, 4, 5]. Disease progression follows a nonlinear and accelerating pattern, leading to implant loss unless properly treated [6, 7]. As such, over the last 30 years, peri‐implantitis has become a major disease faced in dentistry [8].

Considering a cause‐related concept of treatment, current clinical guidelines suggest a stepwise treatment approach for peri‐implantitis management, which should start with the initial nonsurgical step, during which bacterial deposits supra‐ and submarginally are to be removed [9]. If residual signs of disease emerge, a surgical treatment should be planned [9]. Finally, regular supportive care is the last step of the sequential approach [9]. Subsequently, routine clinical assessment of dental implants, including visual examination and regular probing, is essential in monitoring peri‐implant tissue health status [10].

Numerous nonsurgical mechanical treatment protocols, along with various local adjunctive measures (e.g., diode laser, local antimicrobials, probiotics, photodynamic therapy), have shown limited efficacy in establishing peri‐implant tissue health in majority of peri‐implantitis cases [11, 12, 13, 14]. The prescription of systemic antibiotics as an adjunctive therapeutic measure along with nonsurgical treatment was shown to enhance microbiological parameters, whereas ambiguous improvements have been reported for the changes in clinical parameters when compared to the controls [15, 16, 17, 18]. In fact, as recently reported, administration of systemic antibiotics during nonsurgical peri‐implantitis treatment did not prevent the need for additional surgical therapy in the long term [19].

Given the limited reliability of nonsurgical peri‐implantitis treatment, surgical interventions, allowing for direct access to the implant surface, are frequently required [20]. After the removal of biofilm from the contaminated implant's surface, depending on the defect configuration, resective or reconstructive surgical treatment modalities may be implemented [20]. For the treatment of peri‐implantitis‐related bone defects, various reconstruction protocols, including the use of different bone fillers alone (non‐guided bone regeneration (GBR)) or along with the barrier membrane (GBR), have been suggested [11, 21, 22]. Although the most effective reconstructive protocol remains unclear, in terms of resolution of inflammation, the implementation of GBR protocols seem more effective than the non‐GBR treatment approaches [21]. Though a less extensive postoperative soft‐tissue recession may be expected at implant sites treated with adjunctive reconstructive measures, in terms of disease resolution, the clinical benefits of the reconstructive therapy over the non‐reconstructive approaches remain unclear [23, 24, 25].

Two randomized clinical trials (RCTs) with 1‐ to 3‐year follow‐up periods failed to identify any clinical benefits provided by the adjunctive administration of postoperative systemic antibiotics following the surgical non‐reconstructive treatment of peri‐implantitis [26, 27]. Accordingly, the current clinical recommendations do not suggest prescribing systemic antibiotics as an adjunctive therapeutic measure following non‐surgical and surgical peri‐implantitis treatment [9]. Nonetheless, currently, there are no clinical data investigating the potential clinical benefits of the systemic antibiotic prophylaxis and their regime for the surgical peri‐implantitis treatment. In fact, the use of shortened antibiotic protocols have already been investigated in the dentistry field and in several areas of general medicine [28, 29, 30]. Therefore, the aim of the present retrospective analysis was to evaluate the clinical efficacy of systemic antibiotic prophylaxis administered as an adjunct to surgical reconstructive peri‐implantitis treatment. It is hypothesized that oral systemic antibiotic prophylaxis improve clinical therapeutic outcomes (i.e., PD reduction and resolution of BOP) of surgical reconstructive peri‐implantitis treatment.

## 2 Materials & Methods

### 2.1 Study Population

**FIGURE 1:**
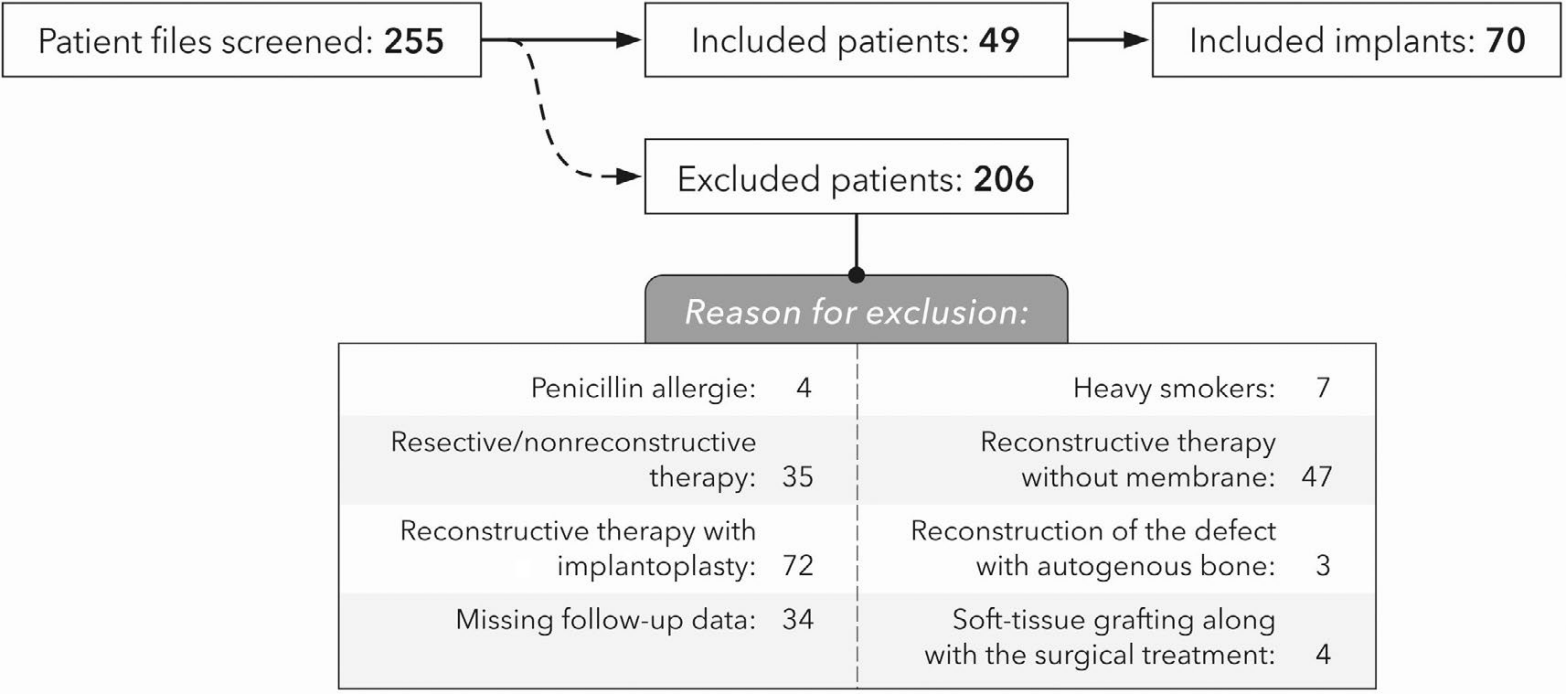
Flow‐chart depicting patient recruitment.

For this retrospective analysis, one investigator (I.S.) screened 255 standardized clinical records of patients who received surgical peri‐implantitis therapy at the Department of Oral Surgery and Implantology, Frankfurt, Germany, between April 2018 and June 2023 (Figure 1). Respecting the inclusion/exclusion criteria, 49 partially edentulous patients with 70 implants that were treated with a surgical reconstructive peri‐implantitis approach were eligible for the analysis. The patient selection process is depicted in a flowchart in Figure 1. The study protocol was in accordance with the Helsinki Declaration, as revised in 2013 and approved by the local ethics committee (registration number: 2023–1571). The study reporting adheres to the checklist items of the STROBE statement [31].

### 2.2 Inclusion and Exclusion Criteria

For patient selection, the following inclusion criteria were defined:

1. Partially or fully edentulous patients rehabilitated with fixed or removable implant‐supported prostheses;
2. Presence of at least one screw‐type, two‐piece bone‐level rough surfaced titanium implant diagnosed with peri‐implantitis;
3. Respective implants treated with reconstructive peri‐implantitis surgical approach;
4. Presence of at least 2 mm of keratinized mucosa;
5. Treated chronic periodontitis and proper periodontal maintenance care;
6. No systemic diseases that could influence the outcome of the therapy (i.e., diabetes [HbA1c < 7], osteoporosis, and antiresorptive therapy);
7. No history of malignancy, radiotherapy, chemotherapy, or immunodeficiency within the last 4 years;
8. Patients attending 6 and 12 months follow‐up appointments;
9. Presence of clinical documentation on bleeding on BOP, Sup, and PDs at the follow‐follow‐up visits; and
10. Nonsmokers or light smoking habits (< 10 cigarettes per day)
11. Patients who did not receive systemic antibiotics within the last 6 months.

For patient selection, the following exclusion criteria were defined:

1. Patients with uncontrolled systemic disorders affecting bone metabolism (diabetes mellitus, osteoporosis, etc.);
2. Patient files missing clinical documentation;
3. Heavy smokers (> 10 cigarettes per day);
4. Patients allergic to penicillin; and
5. Implementation of implantoplasty during surgical reconstructive peri‐implantitis treatment.

### 2.3 Case Definition

Peri‐implantitis was defined as the presence of BOP and/or Sup on gentle probing, increased PDs compared to previous examination, and the presence of radiographic bone loss at the final follow‐up compared to the baseline (i.e., radiographs taken following the placement of the final prosthetic reconstruction) [1].

### 2.4 Treatment Procedures

All patients were subjected to preoperative professional supramucosal/gingival implant/tooth cleaning and underwent a single episode of non‐surgical therapy employing either an Er:YAG laser (KEY3; KaVo, Biberach, Germany) (12.7 J/cm^2^) or an air‐flow device with glycine powder (EMS Airflow). After 2 to 6 weeks, all patients received a standardized surgical reconstructive peri‐implantitis treatment performed by one of four experienced surgeons (F.S., A.R, K.O., and A.B.). In brief, under local anesthesia, full‐thickness mucoperiosteal flaps were raised at vestibular and oral aspects. After removal of granulation tissues with a curette, a titanium brush (Korea Co. Ltd. Gyeonggi‐do, Korea) was used for implant surface decontamination. The intra‐bony three‐ to four‐wall defects with a depth of ≥ 3 mm were filled with either a natural bone mineral (BioOss, Geistlich) or a collagen‐stabilized natural bone mineral (BioOss Collagen, Geistlich) and covered with a native collagen membrane (Bio‐Gide; Geistlich). Mucoperiosteal flaps were repositioned and sutured using non‐resorbable sutures (Cytoplast PTFE, Osteogenics Biomedical, Lubbock, USA) to allow a tension‐free tissue adaptation for the nonsubmerged healing. Postoperative care considered a daily antiseptic mouthwash (0.12% chlorhexidine digluconate solution, Corsodyl, GalxoSmithKline Consumer Helathcare, Buhl, Germany) for 5 days. Sutures were removed after 10 days of transmnucosal healing.

According to the antibiotics regime, patients were subdivided into the following groups:

Pre‐op: patients who received a preoperative single oral shot of antibiotics (2 g of amoxicillin) (Amoxicillin, Aliud Pharma, Laichingen, Germany).

Post‐op: patients who were prescribed with postoperative antibiotics for 3 days (500 mg of amoxicillin, 3x day) (Amoxicillin, Aliud Pharma, Laichingen, Germany).

No‐Ab: patients who received no systemic antibiotics.

### 2.5 Assessment of Clinical Outcome Measures

The following clinical measurements were recorded at six aspects per implant at baseline (i.e., prior to surgery), at 6 and 12 months using a periodontal probe: (1) BOP (presence/absence), (2) plaque (PI) (presence/absence), (3) Sup (presence/absence), (4) mean PD (measured from the mucosal margin to the bottom of the probable pocket), (5) max PD (the deepest PD value at implant site), (6) disease resolution was defined as a combination of the following criteria: PD ≤ 5 mm, ≤ 1 BOP site, and no Sup.

The mean PD reduction from baseline to 6 and 12 months was defined as primary outcome measure. The changes in BOP, Sup, max PD after 6 and 12 months and disease resolution were considered as secondary outcomes.

### 2.6 Statistical Analysis

The statistical analysis was performed using the software programs R (https://www.r‐project.org/, packages “nlme,” “lme4,” and “multicomp”), RStudio (https://posit.co/downloads/) and the R‐scripts from Ref. [31] available at https://github.com/psterzinger/softpen_supplementary.

The BOP (in percentage) represented the proportion of implants/patients with more than one site featuring BOP. The Sup (in percentage) represented the proportion of implants/patients with at least one site with Sup. For the PI, the mean value of the measurements at six sites per implant were estimated. The descriptive data for PI, BOP (in percentage), Sup (in percentage), mean PD and max PD at baseline, after 6 and 12 months following the treatment, the PI, PI reduction (in percentage), BOP reduction (in percentage), Sup reduction (in percentage), changes in PD (mean and max) and reduction of PD (mean and max) (in percentage) occurring after 6 and 12 months were assessed at the patient‐ and implant‐levels. The frequency distribution of residual PDs of 1—3 mm, 4–5 mm and > 5 mm were assessed at baseline, after 6 and 12 months. The BOP/Sup reduction (in percentage) was defined as the proportion of implants/patients that improved to BOP ≤ 1 site or no Sup, respectively over the respective period of time. The PI/mean PD/max PD reduction (in percentage) recorded the proportion of implants/patients that showed an improvement of the PI/mean PD/ max PD values over the respective time period. At the patient‐level analysis, the BOP and Sup were set as 1 if the patient had at least one implant with BOP/Sup > 1 site. The PI, mean PD and max PD for each patient were defined as the mean of the measurements obtained at all the implants exhibited by the patient. Disease resolution was also assessed 6 and 12 months following the treatment at the patient‐ and implant‐levels. The disease resolution at the patient‐level was defined as 1 if all the implants exhibited by the patient displayed disease resolution, and 0 if at least one implant did not fulfill the definition of disease resolution.

Multilevel analyses that account for multiple implants per patient and multiple time points (baseline, 6 months, and 12 months) were conducted. To compare PI, mean PD, and max PD among the groups at each time point, linear regressions with mixed effects were conducted. When the residuals did not meet the assumption of normality, the logarithm of the respective dependent variable was considered. To compare BOP, Sup between the groups at baseline, 6 months, and 12 months; disease resolution at 6 months and 12 months; the reduction of Sup/PI/mean PD from baseline to 6 months and from baseline to 12 months between the groups; and logistic regressions with mixed effects were performed. The linear and logistic regressions with mixed effects considered the patient and the implant as random effects for the implant‐level analysis and the patient as a random effect for the patient‐level analysis. The regressions were followed by general hypothesis testing to ensure the correction of the *p*‐values for multiple comparisons. For the analysis of the Sup (patient level) and BOP/max PD reduction (implant and patient level), maximum softly‐penalized likelihood analysis for mixed‐effects regressions [32] was conducted to ensure the estimates' finiteness. This was followed by Bonferroni adjustment of the *p*‐values. The Shapiro–Wilk‐test was conducted to check normality. The significance level for the analyses was set at 5%.

With a sample size of 70 implants (13, 38, and 19 in the No‐Ab, Pre‐op, and Post‐op groups, respectively), considering one implant per patient, an effect size of 0.6 (Cohen's *w*) can be detected with 80% power when three chi‐squared‐tests are conducted at significance level alpha = 1.7% (correction for multiple testing) to compare the primary variable PD reduction from baseline to 12 months between the groups. The power analysis was conducted with BiAS for Windows (https://www.bias‐online.de/).

## 3 Results

Of the 255 patient files screened, 74 were excluded due to the use of implantoplasty performed along with a reconstructive peri‐implantitis treatment, and 35 were excluded due to the implementation of non‐reconstructive/resective treatment measurers. Thirty‐six patient files were missing follow‐up documentation, and 47 patients underwent reconstructive peri‐implantitis treatment without a barrier membrane. Other reasons for patient extraction are indicated in Figure 1.

Finally, a total of 45 patients exhibiting 67 implants (34 [51%] in the maxilla, and 33 [49%] in the mandible) met the inclusion criteria and were considered for the analysis. Of them, 27 (11 male, 16 female, mean age: 63.78 ± 17.03 years) with 38 implants were allocated to the Pre‐op group, 12 patients (6 male, 6 female, mean age: 63.05 ± 9.48 years) exhibiting 19 implants were assigned to the Post‐op group and the remaining 10 patients (4 male, 6 female, mean age: 69.9 ± 5.82 years) with 13 implants were designated to the No‐Ab group. The majority of all implants (53 implants, 76%) were located in posterior regions, and the rest 17 implants (24%) were placed in the anterior regions (Table 1). Within the first 4 months, one implant in the Pre‐op group was lost; therefore, 37 implants were included in the 6 and 12 month analyses. The survival rates of the implants were 97%, 100%, and 100% in the Pre‐op, Post‐op, and No‐ab groups, respectively.

**TABLE 1:** Descriptive patient and implant‐related data.

	No‐ab	Pre‐op	Post‐op
Patients	10	27	12
Male/female	4/6	11/16	6/6
Age (years; mean ± SD)	69.9 ± 5.82	63.78 ± 17.03	63.05 ± 9.48
Smoking status			
Non‐smokers	10	24	9
Light smokers (< 10 cig./day)	0	3	3
Implants	13	38	19
Implant location Maxilla/mandible	7/6	19/19	10/9
Anterior^a^/posterior	2/11	10/28	5/14
Removed implants	0	1	0

*Note:* Anterior^a^—from canine to canine; SD—standard deviation.

The mean PI, BOP, mean PD, max PD, and Sup values measured at baseline and after 6 and 12 months in the groups are presented in Table 2. The mean PI values were overall low and ranged between 0.3 and 0.4 at the implant‐level estimations and between 0.28 and 0.43 at the patient‐level estimation. At baseline, in the No‐Ab, Pre‐op, and Post‐op groups, 100%, 92.1%, and 94.7% of implants correspond to 100%, 96.3%, and 91.7% patients featured BOP > 1 site, respectively. The Sup was present at 61.5% of implants and 70% patients in the No‐Ab group, 52.6% of implants and 66.7% of patients in the Pre‐op group, and 52.6% of implants and 58.3% of patients in the Post‐op group. The mean PD values in the implant‐ and patient‐level analyses were 5.15 ± 1.57 mm and 5.22 ± 1.67 mm, respectively, in the No‐ab group. The corresponding mean PD values in the Pre‐op group were 5.32 ± 2.06 mm (implant‐level) and 5.46 ± 2.16 mm (patient‐level). In the Post‐ab group, the respective values were 4.28 ± 1.47 mm (implant‐level) and 4.28 ± 1.32 mm (patient‐level). At the implant level, the max PD values in the No‐Ab, Pre‐op, and Post‐op groups were 7.0 ± 1.33 mm, 7.0 ± 2.28 mm, and 6.37 ± 1.77 mm, respectively. The respective measurements in the patient‐level analysis were 6.81 ± 1.55 mm (No‐Ab), 6.58 ± 2.32 mm (Pre‐op), and 5.84 ± 1.32 mm (Post‐op). At baseline and after 6 and 12 months, no significant differences were found among the groups for either of the assessed clinical parameters.

**TABLE 2:** Clinical parameters at baseline, 6 months and 12 months in the investigations groups.

Baseline	No‐Ab	Pre‐op	Post‐op	*p*‐value
Implant‐level				
PI	0.37 ± 0.35	0.34 ± 0.42	0.40 ± 0.41	A:0.97^a^ B:1.00 C:0.87
BOP (percentage of implants with BOP > 1 site)	100	92.1	94.7	A:0.86^a^ B:0.85 C:1.00
Sup (percentage of implants with at least 1 Sup)	61.5	52.6	52.6	A:0.96^a^ B:0.92 C:1.00
PD (mm) Mean ± SD	5.15 ± 1.57	5.32 ± 2.06	4.28 ± 1.47	A: 0.91^a^ B: 0.57 C: 0.12
PD (*n*, %)				
1–3 mm	2 (15.5)	5 (13.2)	5 (26.3)	
4–5 mm	5 (38.5)	14 (36.8)	9 (47.4)	
> 5 mm	6 (46.2)	19 (50.0)	5 (26.3)	
Max PD (mm)	7.46 ± 1.33	7.00 ± 2.28	6.37 ± 1.77	A: 1.00^a^ B: 0.87 C: 0.72
Patient‐level				
PI	0.43 ± 0.35	0.28 ± 0.39	0.39 ± 0.39	A:0.86^b^ B:0.99 C:0.94
BOP (percentage of patients with BOP > 1 site)	100.0	96.3	91.7	A:0.74^b^ B:0.89 C:1.00
Sup (percentage of patients)	70.0	66.7	58.3	A:1.00^c^ B:1.00 C:1.00
PD (mm) Mean ± SD	5.22 ± 1.67	5.46 ± 2.16	4.28 ± 1.32	A: 0.89^b^ B: 0.67 C: 0.14
PD (n, %)				
1–3 mm	1 (10.0)	3 (11.1)	1 (8.3)	
4–5 mm	4 (40.0)	11 (40.7)	8 (66.7)	
> 5 mm	5 (50.0)	13 (48.1)	3 (25.0)	
Max PD (mm)	6.81 ± 1.55	6.58 ± 2.32	5.84 ± 1.45	A: 0.99^b^ B: 0.96 C: 0.76

6‐months	No‐Ab	Pre‐op	Post‐op	*p*‐value
Implant‐level				
PI	0.14 ± 0.21	0.44 ± 0.54	0.38 ± 0.45	A:0.88^a^ B:0.98 C:0.99
BOP (percentage of implants with BOP > 1 site)	0	48.6	21.1	A:0.50^a^ B:0.88 C:0.11
Sup (%)	0.0	13.5	0.0	A:1.00^a^ B:1.00 C:0.76
PD (mm) Mean ± SD	3.21 ± 1.12	3.77 ± 1.66	2.84 ± 0.81	A: 0.73^a^ B: 0.78 C: 0.14
PD (n, %)				
1–3 mm	6 (46.2)	13 (35.1)	10 (52.6)	
4–5 mm	6 (46.2)	19 (51.4)	9 (47.4)	
> 5 mm	1 (7.7)	5 (13.5)	0 (0.0)	
Max PD (mm)	4.15 ± 1.57	4.76 ± 1.59	4.11 ± 1.10	A: 1.00^a^ B: 0.73 C: 0.49
Disease resolution (%)	92.3	45.9	78.9	A: 0.03^a^ B: 0.34 C: 0.37
Patient‐level				
PI	0.17 ± 0.24	0.44 ± 0.57	0.39 ± 0.39	A:0.95^b^ B:0.99 C:0.99
BOP (percentage of patients with BOP > 1 site)	0.0	51.9	33.3	A:0.41^b^ B:0.99 C:0.24
Sup (%)	0.0	18.5	0.0	A:0.72^c^ B:0.54 C:0.54
PD (mm) Mean ± SD	3.06 ± 0.98	3.77 ± 1.12	2.88 ± 0.79	A: 0.72^b^ B: 0.83 C: 0.15
PD (n, %)				
1–3 mm	5 (50.0)	8 (29.6)	7 (58.3)	
4–5 mm	4 (40.0)	17 (63.0)	5 (41.7)	
> 5 mm	1 (10.0)	2 (7.4)	0 (0.0)	
Max PD (mm)	3.73 ± 1.27	4.30 ± 1.65	3.71 ± 1.05	A: 1.00^b^ B: 0.65 C: 0.44
Disease resolution (%)	69.2	31.6	42.1	A: 0.07^b^ B: 0.11 C: 0.99

12‐months	No‐Ab	Pre‐op	Post‐op	*p*‐value
Implant‐level				
PI	0.19 ± 0.31	0.41 ± 0.48	0.25 ± 0.34	A:0.88^a^ B:0.98 C:0.99
BOP (percentage of implants with BOP > 1 site)	30.8	32.4	5.3	A:0.50^a^ B:0.88 C:0.11
Sup (%)	0.0	5.4	5.3	A:1.00^a^ B:1.00 C:0.76
PD (mm) Mean ± SD	3.46 ± 1.54	3.57 ± 1.20	3.15 ± 1.12	A: 0.73^a^ B: 0.78 C: 0.14
PD (n, %)				
1–3 mm	5 (38.5)	16 (43.2)	10 (52.6)	
4–5 mm	5 (38.5)	17 (45.9)	7 (36.8)	
> 5 mm	3 (23.1)	4 (10.8)	2 (10.5)	
Max PD (mm)	5.15 ± 2.41	4.54 ± 1.56	4.05 ± 1.39	A: 1.00^a^ B: 0.73 C: 0.49
Disease resolution (%)	61.5	64.9	84.2	A: 0.048^a^ B: 0.67 C: 0.14
Patient‐level				
PI	0.25 ± 0.34	0.43 ± 0.50	0.25 ± 0.33	A:0.95^b^ B:0.99 C:0.99
BOP (percentage of implants with BOP > 1 site)	40.0	37.0	8.3	A:0.41^b^ B:0.99 C:0.24
Sup (%)	0.0	7.4	8.3	A:1.00^c^ B:1.00 C:1.00
PD (mm) Mean ± SD	3.47 ± 1.58	3.55 ± 1.19	3.16 ± 1.15	A: 0.72^b^ B: 0.83 C: 0.15
PD (n, %)				
1–3 mm	4 (40.0)	12 (44.4)	6 (50.0)	
4–5 mm	4 (40.0)	13 (48.1)	5 (41.7)	
> 5 mm	2 (20.0)	2 (7.4)	1 (8.3)	
Max PD (mm)	4.46 ± 2.58	3.83 ± 1.78	3.29 ± 1.44	A: 1.00^b^ B: 0.65 C: 0.44
Disease resolution (%)	61.5	73.7	89.5	A: 0.10^b^ B: 0.40 C: 0.84

*Note:* A: No‐Ab vs. Pre‐op, B: No‐Ab vs. Post‐op, C: Pre‐Ab vs. Post‐op.

^a^ Implant level: logistic regression with mixed effects.

^b^ Patient‐level: logistic regression with mixed effects.

^c^ Maximum softly‐penalized likelihood for mixed effects logistic regression.

Table 3 represents the change in the PI, BOP, mean PD, max PD, and Sup after 6 and 12 months in all three groups. After 6 months, mean PI values decreased by −0.23 ± 0.34 and − 0.27 ± 0.37 in the No‐Ab group at the implant and patient levels, respectively. The corresponding values were 0.11 ± 0.41 and 0.15 ± 0.43 in the Pre‐op group, −0.01 ± in the Pre‐op group, and − 0.01 ± 0.67 and 0.06 ± 0.54 in the Post‐op group. After 12 months, the mean PI values decreased by −0.18 ± 0.33 (implant‐level) and −0.18 ± 0.35 (patient‐level) in the No‐ab group. In the Pre‐op group, the respective changes amounted to 0.09 ± 0.42 (implant‐level) and 0.15 ± 0.40 (patient‐level). In the Post‐op group, the PI changes after 12 months were −0.16 ± 0.50 (implant‐level) and −0.07 ± 0.50 (patient‐level). The differences among the groups were not statistically significant.

**TABLE 3:** Changes in clinical parameters after 6 and 12 months.

Baseline 6‐months	No‐Ab	Pre‐op	Post‐op	*p*‐value
Implant‐level				
PI	−0.23 ± 0.34	0.11 ± 0.41	−0.01 ± 0.67	
PI dich (percentage of implants with PI improvement)	46.2	16.2	42.1	A: 0.19^a^ B:0.98 C:0.33
BOP (%)	100	43.2	73.7	A: 0.024^b^ B:0.18 C:0.06
Sup (%)	61.5	40.5	52.6	A: 0.45^a^ B: 0.94 C:0.77
PD (mm) Mean ± SD	−1.95 ± 1.77	−1.55 ± 1.72	−1.44 ± 1.66	
PD dich (percentage implants with PD improvement)	92.3	83.8	98.5	A: 0.93^a^ B: 0.99 C: 0.99
Max PD	−3.31 ± 1.49	−2.27 ± 2.19	−2.26 ± 1.69	
Max PD dich (percentage implants with Max PD improvement)	92.3	86.5	94.7	A: 1.00^b^ B: 1.00 C: 0.66
Patient‐level				
PI	−0.27 ± 0.37	0.15 ± 0.43	0.06 ± 0.54	
PI dich (% of patients with PI improvement)	50	18.5	33.3	A: 0.29^c^ B:0.91 C:0.78
BOP (%)	100.0	44.4	58.3	A: 0.054^b^ B: 0.12 C:0.66
Sup (%)	70.0	51.9	58.3	A: 0.71^c^ B: 0.95 C:0.98
PD (mm) Mean ± SD	−2.16 ± 1.57	−1.69 ± 1.88	−1.41 ± 1.55	
PD dich (% patients with PD improvement)	90	85.2	91.7	A: 0.99^c^ B: 1.00 C: 0.98
Max PD	−3.08 ± 1.24	−2.28 ± 2.09	−2.13 ± 1.26	
Max PD dich (percentage patients with Max PD improvement)	100	92.1	94.7	A: 0.84^b^ B: 0.96 C: 1.00

Baseline 12‐months	No‐Ab	Pre‐op	Post‐op	*p*‐value
Implant‐level				
PI	−0.18 ± 0.33	0.09 ± 0.42	−0.16 ± 0.50	
PI dich (% of implants with PI improvement)	46.2	18.9	47.4	A: 0.28^a^ B:0.99 C:0.29
BOP (%)	69.2	59.5	89.5	A: 0.9^b^ B: 0.29 C:0.06
Sup (%)	61.5	48.6	47.4	A: 0.64^a^ B: 0.82 C:0.99
PD (mm) Mean ± SD	−1.69 ± 1.56	−1.75 ± 1.76	−1.13 ± 0.99	
PD dich (percentage implants with PD improvement)	76.9	86.5	94.7	A: 0.92^a^ B: 0.65 C: 0.91
Max PD	−2.31 ± 2.21	−2.49 ± 2.24	−2.32 ± 1.20	
Max PD dich (percentage implants with Max PD improvement)	76.9	89.2	100	A: 0.42^b^ B: 0.18 C: 0.42
Patient‐level				
PI	−0.18 ± 0.35	0.15 ± 0.40	−0.07 ± 0.50	
PI dich (percentage of patients with PI improvement)	50	18.5	41.7	A: 0.29^c^ B:0.99 C:0.49
BOP (%)	60.0	59.3	83.3	A: 1.00^b^ B: 0.42 C:0.29
Sup (%)	70.0	63.0	50.0	A: 0.95^c^ B: 0.79 C:0.95
PD (mm) Mean ± SD	−1.74 ± 1.56	−1.91 ± 1.88	−1.13 ± 1.06	
PD dich (% patients with PD improvement)	80	92.6	91.7	A: 0.79^c^ B: 0.92 C: 1.00
Max PD	−2.34 ± 1.93	−2.75 ± 2.07	−2.55 ± 0.93	
Max PD dich (percentage patients with Max PD improvement)	76.9	92.1	100	A: 0.24^b^ B: 0.18 C:0.60

*Note:* A: No‐Ab vs. Pre‐op, B: No‐Ab vs. Post‐op, C: Pre‐op vs. Post‐op.

^a^ Implant level: logistic regression with mixed effects.

^b^ Maximum softly‐penalized likelihood for mixed effects logistic regression.

^c^ Patient‐level: logistic regression with mixed effects.

After 6 months, 100%/100%, 43.2%/44.4% and 73.7%/69.7% of implants/patients displayed a decrease in BOP in the No‐Ab, Pre‐op, and Post‐op groups, respectively. The difference between the No‐Ab and Pre‐op groups was statistically significant, in favor of the No‐Ab group (*p* = 0.024). After 12 months, 69.2%/60%, 59.5%/59.3%, 89.5%/83.3%, and 69%/58.35% of implants/patients displayed a decrease in BOP in the No‐Ab, Pre‐op, and Post‐op groups, respectively, with no significant difference found among the groups.

Regarding the changes in Sup, after 6 and 12 months, the number of patients and implants featuring Sup decreased by 61.5% and 70% and by 61.5% and 70%, respectively, in the No‐Ab group. The corresponding values in the Pre‐ab group were 40.5% and 51.6% for implants and patients, respectively, after 6 months and 48.6% and 63% for implants and patients after 12 month. In the Post‐op group, the Sup decreased in 52.6% and 58.3% of implants and patents after 6 months and in 47.4% and 50.5% of implants and patients, respectively, after 12 months. The differences among the groups were not statistically significant.

The mean PD reduction per the implant‐level analysis after 6 months was −1.95 ± 1.77 mm, −1.55 ± 1.72 mm, and −1.44 ± 1.66 mm in the No‐Ab, Pre‐op, and Post‐op groups, respectively. The corresponding values per the patient‐level analysis were −2.16 ± 1.57 mm (No‐Ab), −1.69 ± 1.88 mm (Pre‐op), and −1.41 ± 1.55 mm (Post‐op). After 12 months, the mean PD reduction in the No‐Ab, Pre‐ab, and Post‐ab groups were −1.69 ± 1.56 mm, −1.75 ± 1.76 mm, and −1.13 ± 0.99 mm at the implant level, respectively. At the patient level, the vales were −1.74 ± 1.56 mm (No‐Ab), −1.91 ± 1.88 (Pre‐op), and −1.13 ± 1.06 mm (Post‐op). The differences among the groups were not statistically significant.

The max PD reduction after 6 months was −3.31 ± 1.49 mm, −2.27 ± 2.19 mm, and −2.26 ± 1.69 mm in the No‐Ab, Pre‐op, and Post‐op groups, respectively, at the implant level. At the patient level, the respective estimations were −2.16 ± 1.57 mm (No‐Ab), −1.69 ± 1.88 mm (Pre‐op), and −1.41 ± 1.55 mm (Post‐op). Based on implant‐level estimations, after 12 months in the No‐ab, Pre‐op, and Post‐op groups, the max PD was reduced by −2.31 ± 2.21 mm, −2.49 ± 2.24 mm, and −2.32 ± 1.20 mm, respectively. At the patient level, the respective values were −2.34 ± 1.93 mm (No‐Ab), −2.75 ± 2.07 mm (Pre‐op), and −2.55 ± 0.93 mm (Post‐op). The differences among the groups were not statistically significant.

After 6 months, disease resolution was obtained in 92.3% of implants in the No‐Ab group, 45.9% of implants in the Pre‐op group, and 78.9% of implants in the Post‐op group, with a significantly higher difference obtained the between No‐Ab and Pre‐ab groups, in favor of the No‐Ab group (*p* = 0.03). The corresponding estimations at the patient level were 69.2% in the No‐Ab group, 31.6% in the Pre‐op group, and 42.1% in the Post‐op group. The differences among the groups were not statistically significant. After 12 months, 61.5%, 64.9%, and 84.2% of implants in the No‐Ab, Pre‐op, and Post‐op groups, respectively, displayed disease resolution. The disease resolution was significantly more frequently obtained in implants in the Pre‐op group compared to the No‐Ab group (*p* = 0.048). The respective values at the patient‐level estimation were 61.5% (No‐Ab), 73.7% (Pre‐op), and 89.5% (Post‐op). At the patient level, the differences among the groups were not statistically significant.

## 4 Discussion

The present study was aimed at evaluating the clinical effect of oral systemic antibiotics prophylaxis administered as an adjunct to surgical reconstructive peri‐implantitis treatment throughout a 12‐month follow‐up period. Of the included 49 patients, 27 (38 implants) received a single preoperative dose of antibiotics (2 g amoxicillin, Pre‐op), 12 patients (19 implants) were prescribed with postoperative antibiotics for 3 days (Post‐op), and the remaining 10 patients (13 implants) did not receive any systemic antibiotics (No‐Ab). To the authors' best knowledge, this is the first study investigating the potential benefits of different systemic antibiotic prophylaxis regimes following surgical peri‐implantitis treatment.

Based on the findings of the present analysis, surgical reconstructive peri‐implantitis treatment resulted in considerable reductions in mean BOP values, PD, and Sup assessments after 6 and 12 months compared to the baseline. In particular, the mean PD reduction after 12 months ranged between −1.13 and −1.91 mm (patient‐level analysis), which is within the range of the results reported by the previous clinical studies investigating the outcomes of surgical reconstructive peri‐implantitis treatment [23, 33, 34, 35]. The extent of PD decrease was comparable among the groups. After 6 months, a significantly higher BOP reduction was obtained in the No‐Ab group compared to Pre‐op groups at implant‐level, though the differences at patient‐level analysis were insignificant. After 12 months, 60%, 59.3%, and 83.3% of patients in the No‐Ab, Pre‐op, and Post‐op groups, respectively, displayed reduction in BOP, which aligns with the finding of the previous studies reporting on reconstructive peri‐implantitis treatment [14, 23, 24]. At baseline, 61.5% of implants in the No‐Ab group, as well as 52.6% to 52.6% of implants in the Pre‐op and Post‐op groups, featured Sup, which after 12 months was reduced to 0%, 5.4% and 5.3% of implants in the No‐Ab, Pre‐ab, and Post‐ab groups, respectively. Although surgical treatment resulted in considerable BOP and Sup decrease, the changes among the three investigation groups were comparable, thus suggesting that systemic antibiotics prophylaxis may not have a significant impact upon the resolution of clinical inflammatory signs.

Upon further analysis of the present data set, after 12 months, the disease resolution based on the recently suggested definition [1] was obtained at 61.5%, 54.9%, and 84.2% of the implants in the No‐Ab, Pre‐op, and Post‐op groups, in a significant favor of the Pre‐op group compared to the No‐Ab group. The corresponding values at the patient‐level analysis were 61.5% (No‐Ab), 73.7% (Pre‐op), and 89.5% (Post‐op). Although there was a tendency toward a higher number of patients featuring disease resolution in the Post‐op and Pre‐op groups, the differences among the three groups remained insignificant. In corroboration are the results of one recent RCT, which aimed to assess the effects of systemic antibiotics as an adjunctive therapeutic measure on the clinical and radiographic outcomes following surgical resective peri‐implantitis treatment [36]. Specifically, after 12 months, administration of a combination of amoxicillin and metronidazole (Test 1) or phenoxymethylpenicillin and metronidazole (Test 2) for 7 days failed to improve the clinical efficacy of the treatment, as shown by comparable changes in clinical parameters (i.e., PD, BOP, and Sup values) compared to the Control group (placebo) [36]. On the other hand, the radiographic marginal bone‐level stability and consequent disease resolution, defined as presence of PD ≤ 5 mm, BOP ≤ 1 site, no Sup, and absence of additional bone loss > 0.5 mm, were significantly more frequently obtained in both test groups (Test 1: 68% of implants; Test 2: 66% of implants) compared to the Control group (28% of implants) [36]. In further agreement with the present results are the outcomes of another 12‐month RCT, which suggested that the prescription of azithromycin for 5 days (day of surgery +4 days after surgery; 250 mg × 2/day), along with non‐reconstructive peri‐implantitis treatment, failed to improve clinical and radiographic therapeutic outcomes compared to the Control group (i.e., no systemic antibiotics) [26]. Disease resolution (PD ≤ 5 mm, no BOP/Sup, and no additional bone loss) after 12 months was documented for 46.7% of implants in the test groups, as well as for 25% of the implants in the Control group, with no significant difference detected between the groups [26].

It is worth noting that all of the implants included in the present analysis had modified surfaces. In fact, as shown by the finding of one former RCT, the adjunctive effect of systemic antibiotics following non‐reconstructive/resective peri‐implantitis treatment might be influence by the implant surface characteristics [27, 37]. In particular, after 12 months, the adjunctive use of systemic antibiotics (Amoxicillin 2 × 750 mg) for 10 days was associated with significantly higher treatment success (PD ≤ 5 mm, no BOP/Sup, and bone loss ≤ 0.5 mm) at implants with a modified surface (OR, 38.69; *p* = 0.005), whereas at nonmodified surfaced implants, systemic antibiotics did not have an influence upon a successful treatment outcome (OR, 0.27; *p* = 0.506) [37]. The benefits of systemic antibiotics were, however, not be sustained over 3 years, regardless of implant surface characteristics [27].

As for the local application of systemic antibiotics, based on one RCT, the repeated local applications of antibiotics (i.e., minocycline ointment 1, 3, and 6 months postoperatively) along with the systemic amoxicillin therapy thrice per day of 500 mg for 3 days lead to significant benefits in terms of greater mean PD reduction and radiographic marginal bone levels compared to the control implant sites (i.e., mechanical debridement and air‐powder polishing), whereas changes in BOP/Sup were comparable between the test and control groups [38]. Another RCT was conducted to evaluate the effects of local doxycycline application formulated with β‐tricalcium phosphate bone graft [39]. After 6 and 12 months, the test group (i.e., local antibiotics group) showed significantly greater improvements in bone levels than the control group [39]. Nonetheless, although systemic antibiotics showed promising results in term of disease resolution, in line with the current clinical guidelines, there is still insufficient evidence to make any recommendation on the use of local antibiotics as adjuncts in the surgical treatment of peri‐implantitis [9].

As reported by the previous clinical studies, gastrointestinal events were the most frequently reported side effect in the patients administered with systemic antibiotics [36, 37]. The allergic reactions, increase of bacterial resistance, and development of superinfections appear among the most frequent side effects of systemic administration of antibiotics [40, 41, 42]. It should be acknowledged that all of the clinical studies focusing on surgical peri‐implantitis treatment prescribed systemic antibiotics empirically with a wide spectrum of antibiotics or combinations thereof for 5 to 7 days without antibiotic susceptibility testing. As noted above, the overprescription or inappropriate prescription of antibiotics increases the risk for antibiotic resistance and superinfections in susceptible patients that can exacerbate and maintain disease progression [42]. In fact, as shown by the finding of one recent questionnaire‐based study, more than half of the dentist respondents (111/207; 53.6%) reported the use of systemic antibiotics along with the surgical peri‐implantitis treatment [43]. Amoxicillin alone (81%) or in combination with clavulanic acid (56%) was the most common antibiotic of choice prescribed in implant dentistry [43]. Nonetheless, taking into consideration the risk of superinfections, dysbiosis within the microbiome, and antibiotic resistance, the routine use of systemic antibiotics in the treatment of periodontal and peri‐implant diseases is not recommended [44].

Currently it is a question of debate, whether the use of a shortened antibiotic protocols may be an alternative to a full antibiotics regime to reduce the risk of the antibiotics resistance [28, 45]. The goal of prescribing prophylactic antibiotics is to reduce the size of the bacterial load and to subsequently change the characteristics at the operative site during the soft time that host defenses are impaired by the trauma of surgery [46]. In fact, as shown by the studies from the medical field, the use of a single‐dose of preoperative antibiotics may be indeed an alternative to a multiple doses of antibiotics prescribed to up to 5 days [28]. Additionally, in patients with Stage III/IV Grade C periodontitis, a 3‐day systemic administration of amoxicillin and metronidazole adjunctive to subgingival instrumentation was shown to lead to non‐inferior clinical outcomes after 6‐months compared with a 7‐day‐antibiotics protocol [30, 47].

Though the present analysis failed to identify the additional benefits of systemic antibiotics prophylaxis used either as a single‐dose or as multiple‐doses (3 days), when interpreting the findings of the present analysis, it is important to consider the relatively small sample size in the investigation groups, which may have reduced the statistical power to detect significance among the three treatment modalities. Moreover, the short follow‐up period of 12 months might be too short to evaluate the recurrence of peri‐implantitis. Furthermore, the extent of bone loss and distribution of the bone defect morphologies among the three groups could not be assessed due to the retrospective nature of the study, which might potentially influence the obtained outcomes. Also, the absence of radiographic analysis did not allow for the assessment of the potential benefits of systemic antibiotics for the radiographic defect fill/marginal bone stability. In addition, the present study might have profited from the additional microbiological follow‐up analysis, which, due to the study design, was not feasible. Finally, a major limitation of the study was the lack of clear selection criteria which led to the administration of a perioperative systemic antibiotic prophylaxis in respective patients. Finally, a major limitation of the study was the lack of clear selection criteria which led to the administration of a perioperative systemic antibiotic prophylaxis in respective patients. Moreover, the present analysis may not allow for any conclusions regarding the potential benefit of a one‐shot or multiple shots prophylaxis in patients with specific risk profiles. However, it might be speculated that a systemic antibiotic prophylaxis may be particularly indicated in patients featuring Sup at baseline with deep initial PD values [18]. In fact, prescription of adjunctive systemic antibiotics used either as a single or multiple dose showed a tendency toward a higher disease resolution, thus suggesting that their clinical benefits potentially depend on the severity of the disease.

Within the limitations, the present study failed to show additional clinical benefits of pre‐ or postoperative oral systemic antibiotic prophylaxis used along with surgical reconstructive peri‐implantitis treatment.

## Author Contributions

**Ausra Ramanauskaite:** concept, surgeries, data collection, interpretation and drafting the article. **Ioanna Saltzer:** data collection, interpretation, approval of the final version. **Ninad Padhye:** data extraction; **Amira Begic:** surgeries, data collection, approval of the final version. **Karina Obreja:** surgeries, data collection, approval of the final version. **Iulia Dahmer:** statistical analysis, data interpretation, approval of the final version. **Frank Schwarz:** idea generation, concept, data interpretation and drafting the article, approval of the final version.

## Conflicts of Interest

The authors declare no conflicts of interest.

## Supporting information


**Data S1** Supporting Information.

**Data S2** Supporting Information.

## Data Availability

The data that support the findings of this study are available on request from the corresponding author. The data are not publicly available due to privacy or ethical restrictions.
