# Supplementary material for: Systemic Antibiotic Prophylaxis Adjunctive to Surgical Reconstructive Peri‐Implantitis Treatment: A Retrospective Study
Source: Clin Implant Dent Relat Res. 2024 Dec 15;27(1):e13429. doi: 10.1111/cid.13429 (PMC11798903; doi:10.1111/cid.13429)
Supplement: Supplementary file 1 — Data S1 Supporting Information. [file CID-27-0-s002.pdf]

FACHBEREICH MEDIZIN FRANKFURT
GOETHE-UNIVERSITÄT

Ethikkommission des Fachbereichs Medizin der Goethe-Universität
c/o Universitätsklinikum ·Theodor-Stern-Kai 7 ·60590 Frankfurt am Main

Direktor: Poliklinik für Zahnärztliche Chirurgie
und Implantologie J.W. Goethe Universität
Frankfurt am Main
Herr Prof. Dr. Frank Schwarz
Theodor-Stern-Kai 7, Haus 29
60590 Frankfurt am Main

Ethikkommission

Vorsitz:
Prof. Dr. Sebastian Harder

Geschäftsführung:
Dr. Linda Tenbusch

Bearbeitung des Vorgangs:
Dorothea Bittner

04.01.2024

Geschäftsnummer: 2023-1571-Retrospektive Datenauswertung erstberatend
Studientitel: Vergleich zwischen der Verabreichung von präoperativen Antibiotika und keiner Antibiotikagabe bei der Therapie der rekonstruktiven Periimplantitis - Eine retrospektive Studie

VOTUM

Sehr geehrter Herr Professor Schwarz,

vielen Dank für die Zusendung der Studienunterlagen vom 13.11.2023.

Es bestehen keine berufsrechtlichen und berufsethischen Bedenken. **Die Ethikkommission erteilt eine zustimmende Bewertung**.

Die Ethikkommission geht davon aus, dass die Studienleitung und die ggf. beteiligten Doktorandinnen und Doktoranden die einschlägigen datenschutzrechtlichen Vorgaben kennen und beachten.

Seit dem 25.05.2018 gilt die europäische Datenschutzgrundverordnung (DSGVO). Bei der Einsichtnahme der Unterlagen muss darüber hinaus insbesondere § 12 Hessisches Krankenhausgesetz in Verbindung mit § 24 Hessisches Datenschutzgesetz beachtet werden. Wir weisen darauf hin, dass der Studienleiter für die Einhaltung der Datenschutzanforderungen verantwortlich ist. Datenschutzrechtliche Aspekte von Forschungsvorhaben werden durch die Ethikkommission grundsätzlich nur kursorisch geprüft. Diese Bewertung ersetzt mithin nicht die Konsultation des zuständigen Datenschutzbeauftragten.

Geschäftsstelle

Sekretariat:
Zofia de Rooij
Tel.: 069 / 6301-3758
Fax: 069 / 6301-83434

E-Mail: ethikkommission@kgu.de

Mitarbeiter/innen:
Durchwahl

Dorothea Bittner	Tel.: 3889
Sabrina Böttger	Tel.: 80435
Myriam Freund	Tel.: 7239
Angela Heuser	Tel.: 4552
Dr. Marnie Kopp	Tel.: 80436
Viktoria Martynenko	Tel.: 3884
	Fax: 83434

E-Mail: ethikkommission@kgu.de

https://www.kgu.de/ueber-uns/vorstand-des-universitaetsklinikums/dekan/ethikkommission

Lieferadresse:
Ethikkommission des
Fachbereichs Medizin
der Goethe-Universität
c/o Universitätsklinikum
Theodor-Stern-Kai 7
Haus 1, 2. OG, Zi. 207
60590 Frankfurt am Main

Öffnungszeiten f. Anlieferungen:
Montag bis Donnerstag:
09:00 bis 15:30 Uhr
Freitag:
09:00 bis 12:30 Uhr

Bitte tragen Sie die Studie in die gemeinsame Datenbank „Studienkennzahlen“ unter https://ctcn.kgu.de/apps/WebObjects/ST21-productive-DataCapture.woa/wa/choose?customer=ZENT1 ein. Die Eintragung von retrospektiven Studien ist fakultativ.
Beachten Sie, dass Studien außerhalb des Fachbereichs 16 der Goethe-Universität von diesem Erfordernis ausgenommen sind.

Nach Abschluss der Studie bitten wir Sie uns über das Studienende zu informieren und innerhalb eines Jahres nach Studienende einen Abschlussbericht einzureichen. Hierzu sind Sie gemäß der Deklaration von Helsinki verpflichtet.

Sollte die Studie nicht in dem von Ihnen angegebenen Zeitraum beendet werden können, denken Sie rechtzeitig daran eine Verlängerung des Studienzeitraumes in Form einer wesentlichen Änderung zu beantragen.

Mit freundlichen Grüßen

S. Harder

Prof. Dr. med. Sebastian Harder
Vorsitzender der Ethikkommission

Bitte beachten Sie, dass eine Veröffentlichung des Votums oder sonstiger Bescheide der Ethikkommission mit der persönlich eindeutig zuzuordnenden Unterschrift des Verfassers im Internet nicht zulässig ist. Dadurch wird das allgemeine Persönlichkeitsrecht des Verfassers sowie dessen Recht auf informationelle Selbstbestimmung verletzt.

Die Stellungnahme der Ethikkommission erfolgte aufgrund folgender eingereichter Unterlagen:
1) Anschreiben AB_PI_Nov 2023.pdf vom 13.11.2023
2) Antragsformular_AB_PI_November 2023.pdf vom 13.11.2023
3) Studienprotokoll_AB_PI.pdf vom 13.11.2023
4) Antragsbestätigung.pdf vom 13.11.2023
5) Kurzbeschreibung.pdf vom 13.11.2023
6) CV_Schwarz.pdf vom 05.12.2023

Bei mehrfach eingereichten Unterlagen des gleichen Dokumententyps umfasst das Votum nur die zuletzt eingereichte Version.
